# Observations on the Treatment of Scarlet Fever

**Published:** 1886-06-20

**Authors:** 


### OBSERVATIONS ON THE TREATMENT OF SCARLET FEVER.

---

The practical questions to be considered in the treatment of scarlet fever are the grade of temperature, the frequency of the pulse, the extent of pharyngitis, and of cervical adenitis, the degree of vital and nervous prostration, the condition of the renal organs, and the state of digestion. The following formula has been found useful in a high grade of fever and frequent pulse, associated, as it so often is, with nausea and vomiting:

℞. Aq. calcis ........................................ ℥ iij,

Tinct. aconit. rad ................................ gtt. xij.

Sig. One or two teaspoonfuls to a child of one or two years old every two hours. The dose should be administered each time with a small quantity of cracked ice.

In very high grades of fever, when vomiting and nausea are not present, the following antipyretic combination has been resorted to with decided benefit. It not only reduces fever, but acts as a diuretic and diaphoretic also:

℞. Aquæ ............................................ ℥ ij,

Arom. spts. ammon ............................ ʒ iij,

Sodæ bicarb ...................................... ʒ ij,

Acid salicyl ....................................... ʒ ij,

Tinct. aconiti rad .............................. gtt. xij,

Syrup aurantii cort ............................ ℥ i,

Tinct. digitalis .................................. ʒ i.

Sig. Two teaspoonfuls may be given in ice every two or three hours to a child of five years.

For a prolonged case of the pyogenic form of scarlet fever, where there is suppuration in the petrous portion of the temporal bone or some other point as a beginning, the internal use of a combination of quinine, tincture ferri chloridi, and Fowler's solution of arsenic, is superior to all other remedies. Probably of all antipyretics, quinine is the most permanent in its action. We possess, so far, no antidote to the poison of scarlatina. Stimulants should be applied to counteract the effects of that peculiar poison, as in the case of the poisons of venomous reptiles and insects.

℞. Spts. vin. gal .................................... ℥ ij,

Spts. ammon. arom ............................ ʒ ij,

Aq. cinnamo ...................................... ʒ iij,

Syr. simpl .......................................... ʒ iij, is a very convenient form to administer stimulants. Two or three teaspoonfuls of this preparation may be given, diluted, every two or three hours. Iced milk with one third lithia water, and a small amount of bicarbonate of soda, constitutes a nutriment suitable for all ages of scarlet fever.

In some recent cases of the anginose variety of scarlet fever, a solution of 2-per cent. of muriate of cocaine has been applied to the inflamed surface of the pharynx and tonsils by means of a small hand-atomizer. This has been found to relieve hyperesthesia, allay pain and irritation, and reduce the engorgement of the parts. As a means of cleansing and disinfecting the throat, which is always obstructed with accumulating offensive secretions, the following application is beneficial:

℞. Ol. olivæ ........................ ℥ iij,

Vaselin ........................ ʒ i.

Acid, carbol ........................ gtt. iij,

Sod. bicarb ........................ ℈ i,

Sod. boratis ........................ ℈ ij,

Ol. pic. lig ........................ gtt. ij. M.

Sig. To be injected into the nostrils and nasal cavities every two or three hours.

In all cases of nephritis accompanied with dropsical effusion, envelop the body from the armpits to below the hips with spongiopiline, saturated with hot water, frequently renewed, covered with oiled silk. In those cases attended with extensive effusion in the cavities, threatening apnea, scanty, high colored or bloody urine, dry skin, even in a child of four or five years, at least 10 grains of submur. hydrarg., as a preliminary to other treatment, gives an impetus to the secretions which no other agent does.—*Archives of Practice, in Abstract and New Books.*

